# An Image-Based Sensor System for Low-Cost Airborne Particle Detection in Citizen Science Air Quality Monitoring

**DOI:** 10.3390/s24196425

**Published:** 2024-10-04

**Authors:** Syed Mohsin Ali Shah, Diego Casado-Mansilla, Diego López-de-Ipiña

**Affiliations:** 1 DeustoTech, University of Deusto, 48007 Bilbao, Spain; 2 Faculty of Engineering, University of Deusto, 48007 Bilbao, Spain; dcasado@deusto.es (D.C.-M.); dipina@deusto.es (D.L.d.-I.)

**Keywords:** air pollution, image processing, citizen science, synthetic data, data quantification, environmental monitoring

## Abstract

Air pollution poses significant public health risks, necessitating accurate and efficient monitoring of particulate matter (PM). These organic compounds may be released from natural sources like trees and vegetation, as well as from anthropogenic, or human-made sources including industrial activities and motor vehicle emissions. Therefore, measuring PM concentrations is paramount to understanding people’s exposure levels to pollutants. This paper introduces a novel image processing technique utilizing photographs/pictures of Do-it-Yourself (DiY) sensors for the detection and quantification of $PM_{10}$ particles, enhancing community involvement and data collection accuracy in Citizen Science (CS) projects. A synthetic data generation algorithm was developed to overcome the challenge of data scarcity commonly associated with citizen-based data collection to validate the image processing technique. This algorithm generates images by precisely defining parameters such as image resolution, image dimension, and PM airborne particle density. To ensure these synthetic images mimic real-world conditions, variations like Gaussian noise, focus blur, and white balance adjustments and combinations were introduced, simulating the environmental and technical factors affecting image quality in typical smartphone digital cameras. The detection algorithm for $PM_{10}$ particles demonstrates robust performance across varying levels of noise, maintaining effectiveness in realistic mobile imaging conditions. Therefore, the methodology retains sufficient accuracy, suggesting its practical applicability for environmental monitoring in diverse real-world conditions using mobile devices.

## 1. Introduction

Air quality poses a critical global concern, impacting health and the environment across both developed and developing nations. In Europe, particularly in Poland and Italy, air pollution presents a significant public health challenge due to high emissions of hazardous substances, which substantially contribute to disease development and an elevated rate of premature mortality [1]. Similarly, in Delhi, India, the mortality burden from air pollution rose by 47.9% between 2002 and 2019, underscoring the severe impact of deteriorating air quality on public health [2]. Comprised of a complex mixture of particles and gases, air pollution leads to cardiovascular and respiratory issues across all age groups and inflicts extensive environmental damage [3,4,5]. The development of sustainable cities is increasingly dependent on reducing air pollution and enhancing public awareness of this critical challenge. Achieving this goal requires interdisciplinary research and the deployment of smart sensor technologies, such as Internet of Things (IoT) sensors and mobile apps, which report gathered and aggregated data at specific locations [6]. However, while these technologies offer substantial potential for real-time monitoring and increased public engagement, they also face challenges in ensuring the accuracy and privacy of the collected data, besides having associated considerable installation and maintenance costs.

Traditional environmental monitoring systems, while often sparsely distributed and maintained by official institutions and governmental bodies [7], are known for their accuracy, reliability, and adherence to regulatory standards. However, to gather more detailed and localized environmental data, a Citizen Science (CS) approach has started to increasingly be adopted, involving community engagement through the use of low-cost sensors (LCSs) [8]. While this approach promotes collaborative research, allowing communities to participate in formulating research questions and conducting experiments with minimal professional oversight [9], it also faces challenges such as variability in data quality, the need for proper training, and potential biases in data collection by non-expert participants. In recent years, numerous projects have utilized CS to improve public awareness of air pollution. The Smoke Sense Study, initiated by the U.S. Environmental Protection Agency, employs a mobile application to educate the public about the health risks associated with wildfire smoke exposure. While this participatory science initiative effectively uses crowdsourcing to gather data and elevate public awareness, it also faces challenges such as ensuring the accuracy and reliability of the data collected by participants [10]. Similarly, the COMPAIR project, a European endeavour, has utilized Citizen Science to track and mitigate air pollution in various cities including Athens, Berlin, and Sofia. By deploying affordable sensing technology, COMPAIR involved local communities in the collection and analysis of data concerning air quality and vehicular traffic. However, the project encountered obstacles related to maintaining sensor calibration, dealing with data variability, and engaging a consistent participant base across diverse urban settings [11].

In a similar vein, the SOCIO-BEE project also utilizes Citizen Science to engage communities in tracking environmental pollution, leveraging wearables and drones to collect data more dynamically in Maroussi (Greece), Ancona (Italy) and Zaragoza (Spain). While SOCIO-BEE promotes community involvement and environmental awareness, it encounters parallel hurdles such as ensuring sustained participant engagement and managing the reliability of the data collected from its innovative technological applications.

Both the COMPAIR and SOCIO-BEE projects underscore the potential and complexities of using Citizen Science to address urban air pollution [12]. Similarly in Krakow, Poland, the deployment of low-cost sensors (LCSs) for air quality monitoring serves as a prominent example of employing spatio-temporal models to promote sustainable urban development. This research illustrates how these sensors are strategically placed throughout the city to collect detailed temporal and spatial data on air pollution levels. This method allows for detailed monitoring and analysis, providing critical insights supporting the city’s energy transition and sustainable development strategies. The effectiveness of this approach in Krakow showcases the potential for similar applications in other urban settings, emphasizing the role of innovative technologies in addressing environmental challenges [13]. In the Tianjin–Hebei region of China, the deployment of spatio-temporal models and networks of LCSs has substantially contributed to advancing sustainable development and facilitating the energy transition [14]. In Taiwan, community-based sensing projects have established open frameworks for monitoring fine particulate matter. These initiatives encourage grassroots participation and enhance data accuracy through anomaly detection [15]. Cagliari, Italy, has also implemented a social IoT strategy to advance its monitoring capabilities of traffic and pollution. This system utilizes modern sensors and wireless networks to support strategic planning and real-time monitoring of vehicular emissions [16]. Such advancements are crucial for developing smarter, more sustainable urban environments. Furthermore, it is essential to enhance environmental awareness among citizens to effectively mitigate the adverse impacts of air pollution on human health and urban sustainability [17].

These initiatives demonstrate how data collected by citizens complements official environmental monitoring. Furthermore, citizen-led projects in Bristol have utilized low-cost sensors for diverse environmental monitoring activities, including climate change and noise pollution, transforming participants into active collaborators who contribute throughout the research process from problem identification to results dissemination [18]. These initiatives not only collect crucial environmental data but also involve communities directly, promoting a deeper understanding of environmental issues and motivating proactive engagement in sustainability efforts. For all of these uses worldwide, the World Meteorological Organization (WMO) provides guidance for using an LCS to monitor air pollution, emphasizing the need for accurate calibration and validation against higher-grade sensors. This ensures reliable data collection, especially in varying environmental conditions. The WMO’s guidelines advocate integrating LCSs into broader monitoring networks to expand coverage and enhance the granularity of air quality assessments. Such integration aids in informed decision-making for environmental policy and public health interventions [19]. Something of paramount importance in the use of an LCS is quantifying uncertainty. Thus, using quality assessment methods and performance metrics to explain to final users how reliable LCSs can be in terms of the units and data the sensors provide. Such confusion, if not well addressed by vendors or manufacturers, can also lead to erroneous decisions in public policy, eroding public trust in air quality data and the decisions based on them, which is a hot topic in evidence-based policymaking using citizens’ data (in the Discussion Section, we provide the limitations of the presented approach in terms of correlation with reference stations). In essence, Citizen Science approaches significantly expand the scope of environmental monitoring and deepen the public’s involvement in scientific research, ultimately facilitating a broader understanding of environmental issues and encouraging active participation in sustainability practices.

**Figure 1:**
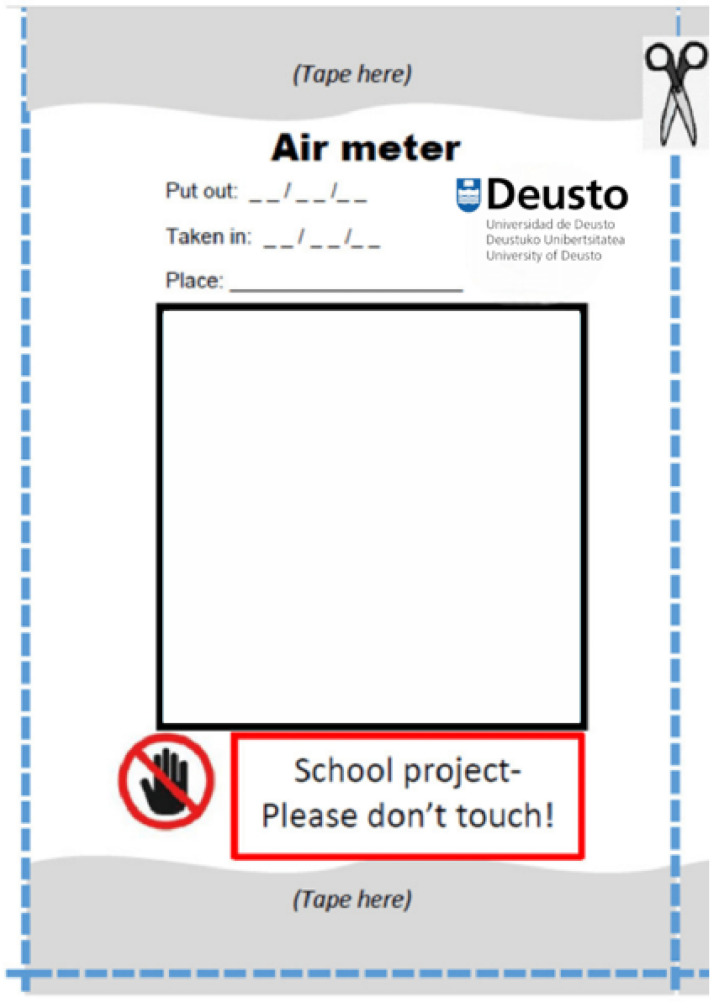
Air meter to monitor dust levels by covering it with a layer of petroleum jelly. Paper covered with a layer of petroleum jelly retains airborne particles which stick to the adhesive material [21].

This paper describes how citizen participation is obtained to capture pictures of air pollution samples using a DiY sensor (see Figure 1). The size of the paper sensor is 6 cm by 6 cm. However, in CS projects there is an inherent challenge of data scarcity due to the variability in sample collection, usually related to the participants’ level of engagement [20]. To tackle this issue, a synthetic PM data generation algorithm is developed that significantly increases the collected dataset. Therefore, facilitating rigorous testing and evaluation of image processing techniques without the need for extensive real-world data collection.

For the automatic generation of images of the DiY sensor needed in this study, a systematic method was employed that ensures the synthetic data closely mirror real-world conditions. The process was initiated by setting precise parameters for the picture. These included image resolution, image dimension, and PM airborne particle density. Each image generated was carefully designed to include a diverse mixture of $PM_{1}$, $PM_{2.5}$, and $PM_{10}$ particles, with sizes ranging from 1 to 10 mm. This diversity reflects the varied particulate matter typically found in urban and rural environments. The image generation algorithm was designed to randomly distribute these particles across different images, thereby ensuring a variety of scenarios could be tested. Furthermore, to add fidelity to the image processing algorithm, three types of noise were introduced: (a) Gaussian noise to replicate the electronic noise present in digital imaging [22]; (b) focus blur to mimic the effect of out-of-focus optics [23]; and (c) white balance adjustment to simulate varying lighting conditions typically encountered in smartphone photography [24].

## 2. Literature Review

The increasing need for efficient air quality monitoring has stimulated the development of cost-effective methods that can be implemented on a large scale. Citizen Science approaches, which utilize the collective efforts of community participants, have notably gained prominence. While these approaches are lauded for democratizing science and allowing broad participation, they also face challenges such as data reliability and the need for rigorous validation against standard environmental sensors. This literature review examines the usage of low-cost sensors (LCSs), specifically tailored for Citizen Science projects aimed at monitoring air pollution related to airborne particulate matter, which includes dust, dirt, soot, smoke, and liquid droplets emitted into the air. The following sections discuss the existing research, focusing on technological developments, methodological challenges, and their implications for public health and environmental policy.

### 2.1. Sources of Particulate Matter

Particulate matter comprises both liquid and solid particles found in the air and is categorized into primary and secondary types [25]. Primary particulate matter is directly emitted into the atmosphere, while secondary particulate matter forms through atmospheric chemical reactions [26]. The main components of particulate matter include inorganic ions, with additional substances such as organic and elemental carbon metals. Organics derived from volatile organic compounds (VOCs), along with sulfates and nitrates, serve as typical examples of secondary particulate matter. Predominant human-made sources vary widely, but notably include the combustion of biomass and fuels. A significant portion of particulate matter originates from the burning of fuel, particularly from vehicle and traffic emissions, highlighting a strong link between particulate matter levels and traffic activity. Furthermore, industrial urban areas with significant fossil fuel burning often exhibit elevated particulate matter levels. Traffic emissions largely influence urban air quality, which is also indirectly impacted by photochemical reactions [27]. In urban settings, factors such as intersections, busy streets, and tall buildings contribute to poor air circulation and the accumulation of primary air pollutants. Consequently, there is a high variability in exposure to primary particulate matter in urban locales, while secondary pollutants tend to have a regional spread influenced by weather conditions. Recent studies provide valuable insights into the regional variations in air pollution sources and their quantifiable impacts on air quality. For example, a study from Lahore, Pakistan, characterizes the city’s air pollution through real-time data analysis, identifying significant contributors such as vehicle emissions and industrial activities. It was found that vehicles alone contributed to 36% of $PM_{2.5}$ pollution, with other sources like industrial emissions and biomass burning also playing critical roles [28]. In [29], a comprehensive analysis of particulate matter (PM) sources was provided across several major cities, including Barcelona, Zurich, London, and Helsinki. In Barcelona, it was found that traffic emissions significantly contribute to PM levels, particularly due to the high density of vehicles in the urban core. Zurich, whilst also affected by traffic, showed a notable influence from residential heating systems during the winter months. In London, besides traffic, industrial emissions and international shipping from the Thames contributed to the city’s PM levels. Helsinki, experiencing colder climates, had distinct PM sources primarily from residential wood burning, which is used extensively for deicing during long winter periods.

### 2.2. Classification of Particulate Matter

Particulate matter is categorized by size, specifically its aerodynamic equivalent diameter (AED), which determines its atmospheric travel distance and potential to penetrate the respiratory system [26]. Fine particulate matter, referred to as $PM_{2.5}$, possesses an aerodynamic diameter of up to 2.5 mm. This small size enables it to travel distances up to 1000 km [27]. The formation of $PM_{2.5}$ typically results from the chemical conversion of nitrogen oxides, sulfur dioxide, and organic compounds during the combustion of substances like oil, coal, and gasoline. Coarse particulate matter, or $PM_{10}$, with an AED of 10 mm, can travel up to 10 km [25,27]. During dust storms, medium-sized particles with diameters ranging from 16 to 31 mm can travel up to 1600 km [30]. $PM_{10}$ is composed of both newly suspended and resuspended soil particles, as well as industrial dust. Particle classification by number distribution is based on count, whereas mass distribution classifications depend on the mass of the particles at each size [26].

### 2.3. Particulate Matter Detectors and Limitations

The World Meteorological Organization (WMO) offers detailed guidance on improving air quality monitoring by integrating traditional reference stations with low-cost sensors (LCSs). These reference stations are equipped with high-accuracy instruments critical for calibrating and validating LCS data, ensuring their reliability for precise environmental assessments. The application of image processing for identifying dust particles marks a significant advancement in environmental science, moving beyond the slow and often imprecise manual techniques used for counting dust particles. Although image processing is useful for tracking dust emissions from vehicles, it sometimes misses the detailed detection and measurement of particulate matter. Importantly, while reference stations based on gravimetric measurements provide exact data, their high cost limits widespread deployment. This highlights the essential role of LCSs as a cost-effective solution in expanding air quality monitoring to diverse locations.

Table 1 provides a comparative overview of common PM detection techniques, highlighting their benefits and drawbacks [31,32,33]. A more updated and comprehensive review can be found in [34]. The Tapered Element Oscillating Microbalance (TEOM) and Beta Attenuation Monitors (BAMs) are recognized for delivering highly accurate real-time data, making them invaluable in scenarios where precision is crucial. However, these systems require substantial infrastructure, including heaters and periodic maintenance, which not only makes them large, heavy, and costly but also limits their deployment in resource-constrained settings. The Black Smoke method offers, in contrast, ease of use and low maintenance requirements, making it suitable for simpler, less critical applications. However, it lacks the precision of the TEOM and BAM and its performance can be significantly influenced by environmental factors. Optical analyzers, on the other hand, offer quick, lightweight solutions capable of measuring multiple particle sizes simultaneously, which provides flexibility and ease of deployment. Yet, their accuracy is also impacted by variations in particle shape and environmental conditions, which can pose challenges in data consistency and reliability.

Similarly, Table 2 lists various sensors used for measuring PM concentrations, specifically $PM_{2.5}$ and $PM_{10}$, categorized by their sensing modules, measurement techniques, and cost [35]. For $PM_{2.5}$, the BAM-1020 Beta Attenuation Measurement Module and Aerocet 831 aerosol mass measurement module use advanced techniques like beta attenuation and light scattering, respectively, but are categorized as high-cost solutions. More affordable alternatives like the OPC-N2 particle sensor and $PM_{2.5}$ Unit utilize light scattering and light obscuration, offering average- to low-cost options. Similarly, for $PM_{10}$, the 602 Betaplus and Aerocet 831 continue to use high-cost techniques, whereas more economical choices like the OPC-N2 and Gp2y1010au use light scattering and light obscuration, respectively, to detect particulate matter at a lower cost.

In a Citizen Science project conducted in Oslo, as detailed in [21], primary school students engaged in air quality monitoring using paper sensors coated with petroleum jelly. These sensors were placed in accessible outdoor areas such as balconies and trees for one week to collect particulate matter. The students manually counted the accumulated particles on the sensors post-exposure with the naked eye technique or using a magnifying lens. While this method served as an educational tool and actively engaged students, it faced accuracy challenges attributed to the subjective nature of visual estimations and inconsistencies in particle adhesion. To improve the accuracy and consistency of PM data, Syed Mohsin et al. [37] introduced an image processing technique for automated particle counting. This technological advancement aimed to minimize human error and variability inherent in manual counting methods, thereby enhancing data reliability and repeatability. The application of image processing in this context not only addressed the methodological limitations but also scaled up the potential of Citizen Science projects in educational environments to produce scientifically reliable data. However, the technique suffered from real-world data scarcity to ensure the effectiveness and testing of the method under several conditions. This paper resolves this issue by providing a synthetic image generation system of polluted images that will then be analyzed to test the efficacy and performance of the detection technique.

## 3. Overview of the Proposed Approach

The proposed approach is divided in two parts: (1) the overall particle detection system that was introduced in [37]; (2) the synthetic image generator, which is the main contribution of this article.

### 3.1. PM Detection System for DiY PM Sensor’s Pictures

**Figure 2:**
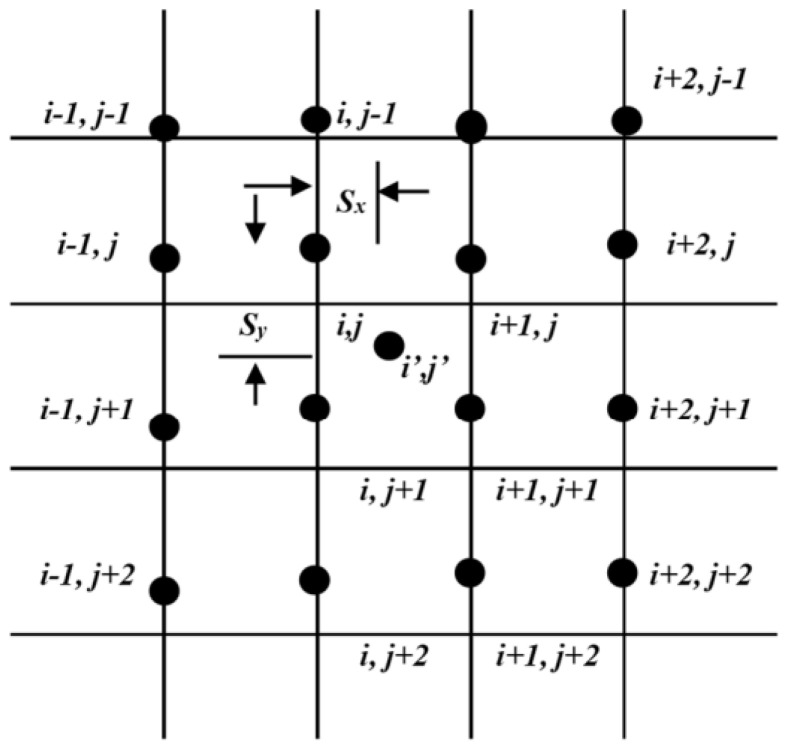
Bicubic interpolation at position (i’, j’) [38].

In the field of machine learning, datasets related to images are typically originated in a raw format looking first for the region of interest (ROI). Then, they usually need an initial preprocessing step. After this initial processing, the next step involves the extraction of pertinent features from the preprocessed data. Once these features have been identified, the process advances to the detection of the source of interest or classification. In this paper, this is $PM_{10}$ particulate matter. However, as airborne particles are very small, some digital image augmentation techniques are needed. Resolution in pixels is given by the metadata of the image. With this information, pixel size to real centimetre (cm) size can be established because the paper DiY sensors of Figure 1 have a square frame of known size in cm (pixel-to-cm conversion can be found in the Appendix A of this paper). To do this, image interpolation is proposed. This is a process that seeks to estimate the intensity of a pixel by considering the values of surrounding pixels based on their proximity [38]. This technique is crucial when resizing or resampling images to align with the requirements of a transmission channel, or to ensure the final image is displayed without perceptible loss in quality. Bicubic interpolation calculates the value of a pixel by taking the weighted average of the 16 nearest neighbouring pixels. This method yields smoother outputs that more accurately represent the original image. Upscaling by bicubic interpolation was employed to achieve a resolution of 1 pixel per micrometre, facilitating the accurate computation of particle diameters from pixels to micrometres. This method enhances image resolution while maintaining the integrity of particle shapes, essential for precise size measurement (extended information about the method can be found in the Appendix B of this paper). Figure 2 shows the bicubic interpolation.

**Figure 3:**
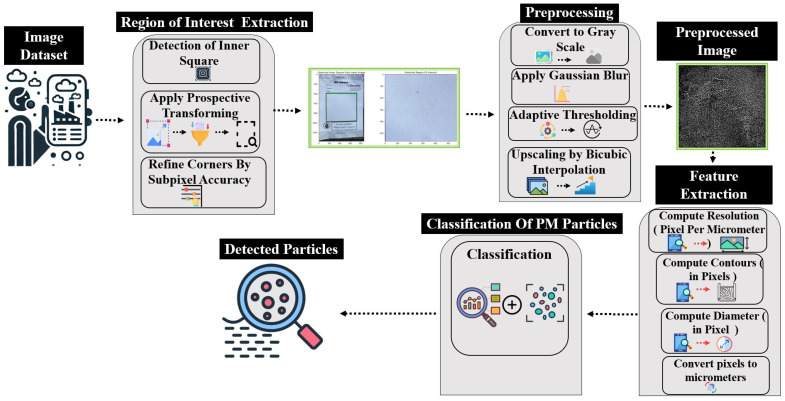
Flow diagram of proposed methodology.

In [37], it was shown that image processing techniques can accurately count particulate matter (PM) particles using a clustering algorithm. Figure 3 demonstrates the basic steps involved in the detection and counting of particles.

### 3.2. Synthetic Image Data Generation

The synthetic image data generation presented in this paper plays a crucial role in enhancing machine learning models by providing diverse and extensive datasets, especially when real data are scarce or sensitive. This method supports the development of robust algorithms by enabling training on a wide range of controlled scenarios and variations, thus mitigating overfitting and improving generalization. Moreover, synthetic data aids in maintaining privacy and ethical standards by circumventing the direct use of real, potentially sensitive, data. In the proposed method, different input parameters are utilized like image height, image width, resolution, and number of particles to replicate the real-world images of particulate matter.

**Figure 4:**
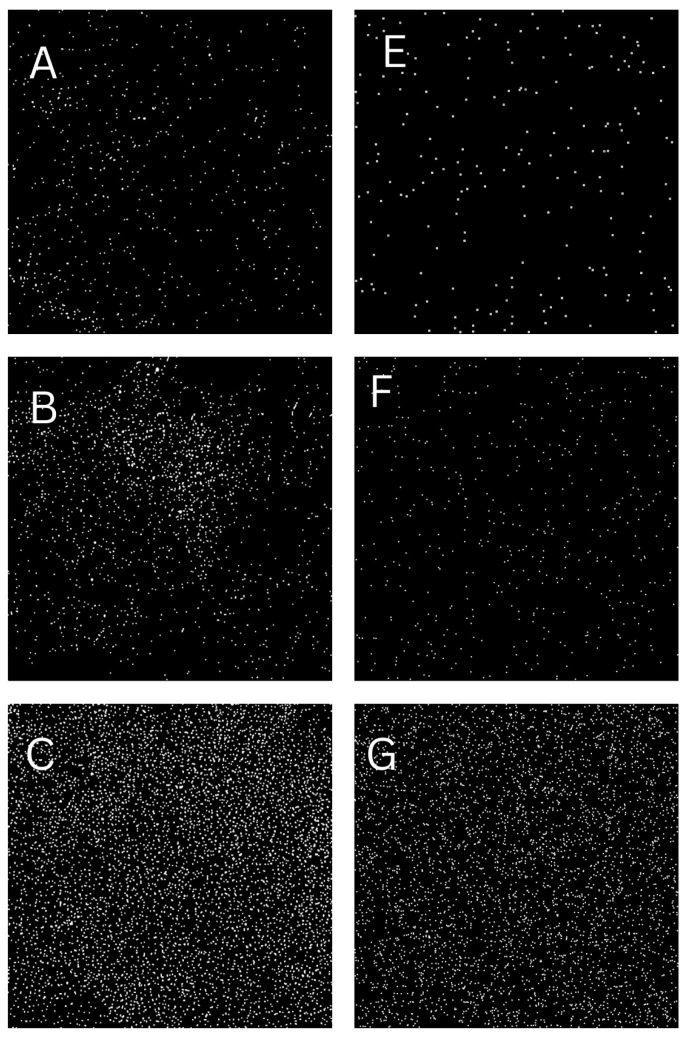
Image sample (**A**–**C**) are samples gathered by [21] and (**E**–**G**) samples are synthetically generated images by proposed method.

**Figure 5:**
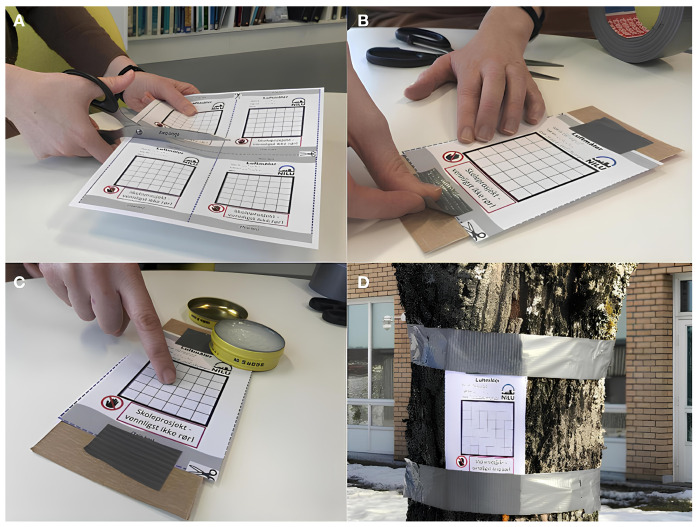
(**A**) Once the air meter has been printed, students can proceed to cut it out. (**B**) The air meter is then affixed to a milk carton using silver duct tape. (**C**) A fine layer of petroleum jelly is applied to the surface using a finger. (**D**) Finally, the air meter is mounted outdoors with the help of silver duct tape [21].

This method produces a diverse set of synthetic images (Figure 4) that closely mimic the variety found in natural imagery, such as the ones that citizens could collect in real life with the DiY sensor after a period of exposure to air in traffic lights or trees. Figure 5 shows how to create the DiY sensor ready to obtain airborne particles after a period of exposition.

The particle sizes, measured in micrometres, indicate the fineness of the dust particles. In our synthetic images, particles with diameters of $PM_{1}$, $PM_{2.5}$, and $PM_{10}$ micrometres were used. Resolution, measured in pixels per micrometre, determines the detail of the dust particles, and in this case, was set at 1 pixel per micrometre. Unlike real-world data, synthetic data generated from these images are not constrained by privacy and ethical considerations, rendering them a more accessible and adaptable resource for research. The ability to modify various parameters enables researchers to tailor these settings, thereby facilitating the creation of synthetic images that accurately simulate real-world scenarios involving dust particles. Table 3 shows us the input parameters for the synthesis of synthetic data samples.

### 3.3. Adding Noises In Synthetic Images

**Figure 6:**
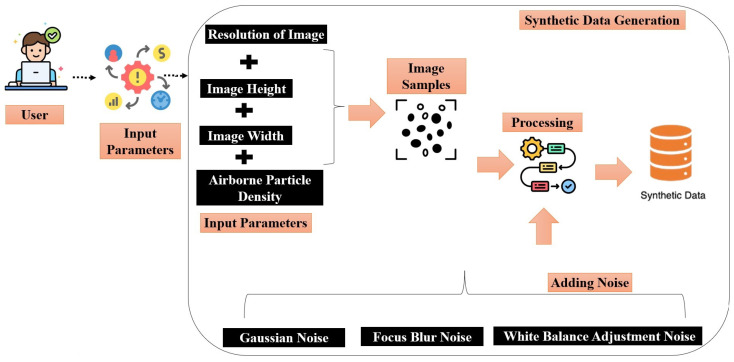
Flow diagram of generation of synthetic data samples and addition of noise.

In the realm of digital image processing, the creation of synthetic images with embedded noise patterns serves as a critical technique for testing and enhancing image analysis algorithms to replicate real-world scenarios. The proposed method is designed to simulate environmental particulate matter within these images. By adjusting parameters such as image width, height, and resolution, it is possible to control the distribution and intensity of these particulate patterns meticulously. Moreover, the image-generating technique mimics the real-world use of digital cameras. For instance, users might use different smartphone cameras and resolutions, paper sensors might have been subject to distortion during their period of exposure due to varying climate conditions, and so on. As such, in the process of enhancing the realism of synthetic images, three distinct types of noise disturbances were introduced. Specifically, Gaussian noise, focus blur, and white balance adjustment. According to Bielova et al. [39], by integrating these noise types, the correspondence of the generated images to real-world captured images is enhanced. Figure 6 shows us the flow diagram of synthetic data generation and the addition of noise.

#### 3.3.1. Addition of Gaussian Noise

Gaussian noise is a statistical noise with a probability density function equal to the normal distribution, also known as the Gaussian distribution. In terms of image processing, this type of noise can be characterized by its mean and variance and is often used to model the random electronic noise present in physical devices such as cameras. The importance of Gaussian noise lies in its capacity to mimic this inherent randomness observed in real-world photographic images, thereby providing a crucial tool in the realm of image synthesis [40]. By adding Gaussian noise to synthetic images, the subtle variability found in photos taken with phone cameras can be replicated, enhancing their realism. This technique allows for synthetic images to better match the statistical properties of real-world data, making them more useful for training and evaluating image processing algorithms.

#### 3.3.2. Addition of Focus Blur Noise

Focus blur noise, often referred to simply as blur, results from a decrease in the sharpness of an image, typically mimicking out-of-focus optics. This type of noise is significant in image processing as it closely resembles common photographic anomalies caused by imperfections in camera focus mechanisms or motion blur, which are frequent in smartphone photography [41]. The introduction of focus blur noise to synthetic images serves to mirror these real-world conditions, enhancing the visual authenticity of the images. By systematically adjusting the focus area and blur strength parameters in a sequential manner across various synthetic images, a spectrum of realistic scenarios was effectively simulated. This adjustment not only improves the realism of the images but also tests the robustness and effectiveness of our image processing algorithm in counting particulate matter under practical conditions commonly encountered in everyday life.

#### 3.3.3. Addition of White Balance Adjustment Noise

White balance adjustment noise refers to variations in color temperature that affect the color accuracy in digital images. This type of noise is critical in the context of image processing because it simulates the challenges encountered in maintaining color fidelity across different lighting conditions, a common issue while taking images by smartphones [42]. The incorporation of white balance noise into synthetic images is executed by altering the temperature parameter in ascending order, which effectively replicates the shifts in hues observed in real-world imaging. This practice enhances the synthetic images’ realism, making them more representative of the variances seen when images are taken under diverse lighting environments. Such adjustments are particularly valuable for testing and improving the performance of our image processing algorithm designed to correct or adapt to color discrepancies in digital images.

## 4. Experimental Outcomes Across Different Experimental Settings with Noise

In this section, the outcomes of the research are discussed and the proposed methodology is elucidated. Following the preprocessing and feature extraction stages, particulate matter (PM) was categorized using a deterministic method by using the pixel-to-cm formula. This classification was based on particle sizes; if their diameter measured 10 mm or smaller then they were categorized as $PM_{10}$. The effectiveness of this approach was assessed by evaluating the accuracy. This section describes the metrics used for calculating the accuracy of the system against reference values of airborne particles and proposes five experiments to assess the impact of noise(s) in the synthetic data generation technique.

### 4.1. Accuracy

To assess the effectiveness of the proposed system, accuracy as a performance metric was employed. Accuracy was determined by the percentage of samples that were correctly classified. These metrics collectively provide a comprehensive evaluation of the system’s performance in various operational scenarios. In the field of predictive modeling, accuracy serves as a crucial metric, representing the proportion of correct predictions made by a model out of all predictions attempted. For the proposed model, accuracy is specifically considered as an indicator of the model’s effectiveness in correctly classifying the particulate matter. This metric is quantitatively derived using Equation (1), which calculates the percentage of accurate predictions. This approach provides a straightforward and robust measure of the methodology’s performance, crucial for evaluating its applicability in real-world scenarios where accurate classification of particulate matter is essential.

(1) $$\mathrm{Accuracy}=\left(\frac{\mathrm{Number}\;\mathrm{of}\;\mathrm{Detected}\;\mathrm{Particles}}{\mathrm{Actual}\;\mathrm{Number}\;\mathrm{of}\;\mathrm{Particles}}\right)\times 100$$

where the *Number of Detected Particles*  may be recorded with or without the influence of noise. Images containing a pre-known quantity of $PM_{10}$ particles were tagged, establishing a definitive actual count. This controlled setup allows us to precisely gauge the effectiveness of our detection algorithm in identifying the correct number of $PM_{10}$ particles.

### 4.2. Reference for Classification

This systematic classification of airborne particles using images from DiY sensors allows for a precise analysis of particle size distribution, crucial for understanding the environmental impacts and health implications associated with particulate matter. Classification criteria can be found in the Appendix A of this paper. The different pollution levels can be assessed against the reference for this paper, which is the dust scale by [21], shown in Table 4.

In the following, the effect of the different noises and their impact on performance are evaluated.

### 4.3. Examining the Effects of Adding Gaussian Noise in Synthetic Data Samples

**Figure 7:**
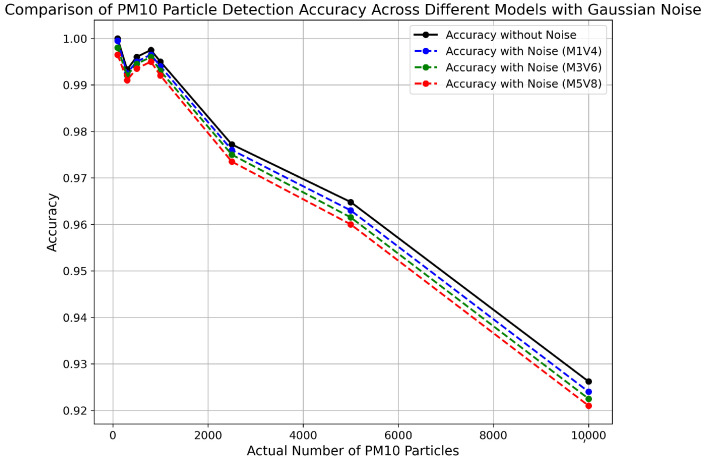
Comparison of accuracy of proposed methodology with addition of Gaussian moise for different input mean and variance values.

The addition of Gaussian noise over synthetic images, with a mean value in the range between 1 and 5 and a variance in the range between 4 and 8, reveals consistent accuracy across various $PM_{10}$ particle concentrations. For particle counts, ranging from 100 to 800, accuracy maintains high levels, indicating the robustness of the $PM_{10}$ detection algorithm even in the presence of simulated noise. This initial variation establishes a baseline for comparing the influence of different Gaussian noise parameters in subsequent variations, providing key insights into the image processing technique’s potential for real-world applications, particularly in engaging citizen scientists in environmental monitoring projects with smartphones with different quality cameras. The analysis of Gaussian noise’s impact on $PM_{10}$ particle detection underscores the robustness of the detection algorithm across varying noise intensities, essential for simulating realistic mobile imaging conditions. Figure 5 shows minimal variation in accuracy metrics, maintaining applicable scores. These results are vital for applications in Citizen Science projects, demonstrating that the image processing algorithm’s performance is unaffected by common fluctuations in background noise, thus ensuring reliable data collection from non-expert users in real-world environments. This consistency not only validates the image processing algorithm’s utility in diverse environmental conditions but also strengthens its role in enhancing participatory scientific research, where community-generated data are crucial. Figure 7 shows us the comparison of the accuracy of proposed method with the addition of Gaussian noise for varying input parameters.

### 4.4. Examining the Effects of Adding Focus Blur Noise in Synthetic Data Samples

**Figure 8:**
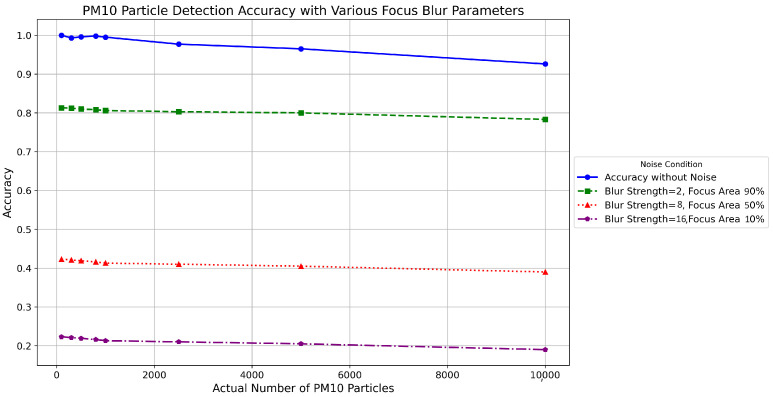
Comparison of accuracy of proposed methodology with addition of focus blur noise for different input blur strength and focus area values.

**Figure 9:**
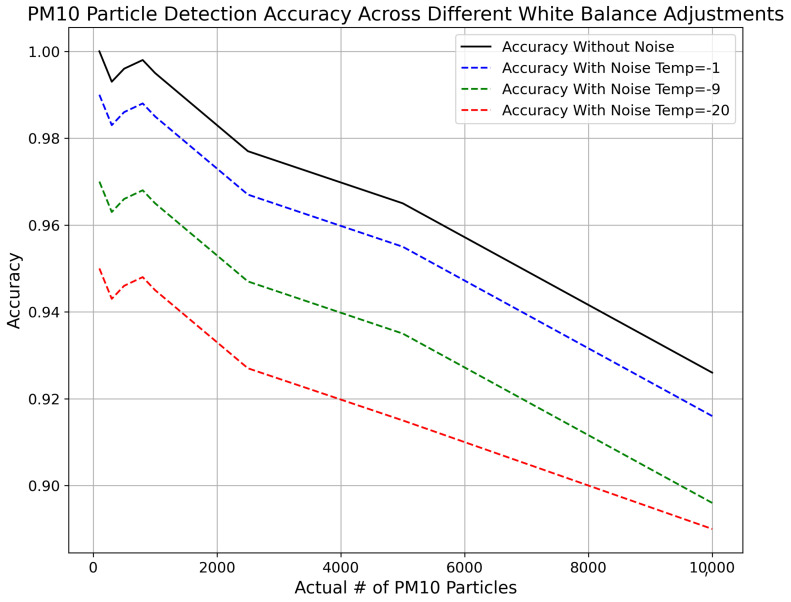
Comparison of accuracy of proposed methodology with addition of white balance noise for different input values.

**Figure 10:**
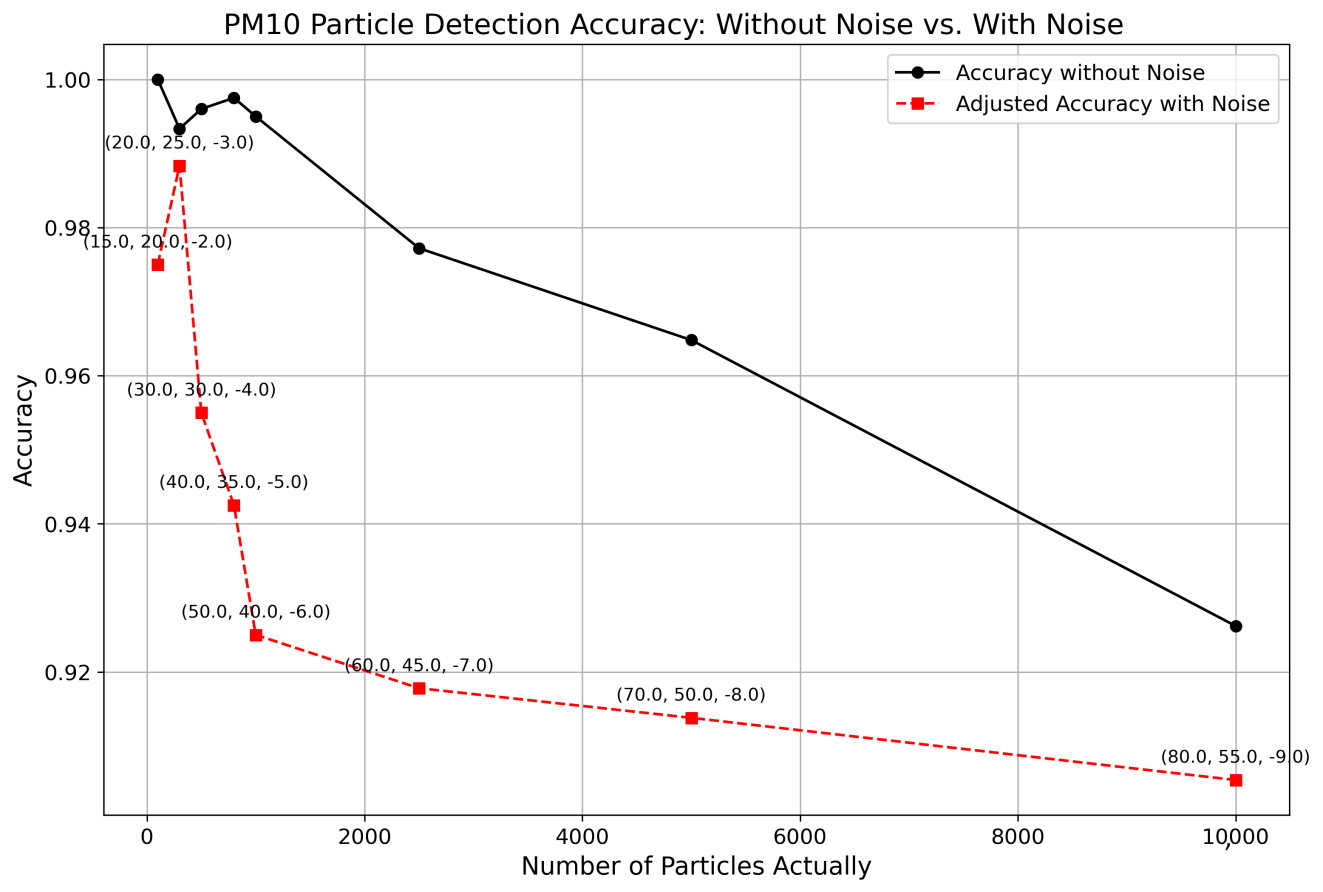
Comparison of accuracy of proposed methodology with addition of Gaussian noise and white balance noise for different input values.

**Figure 11:**
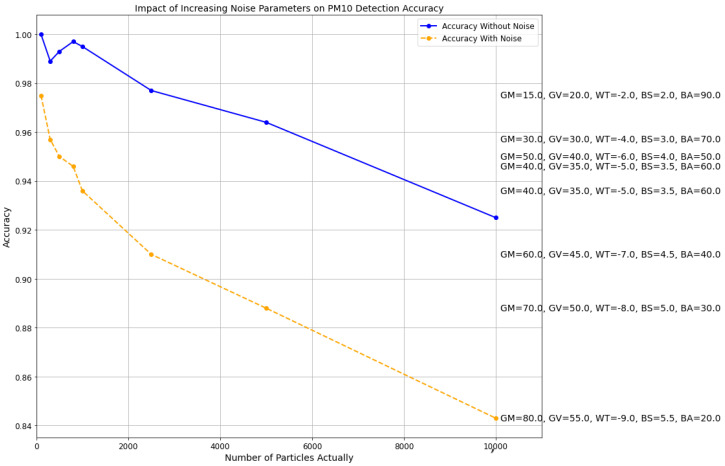
Comparison of accuracy of proposed methodology with addition of Gaussian noise, focus blur, and white balance noise for different input values.

This section assesses the impact of focus blur noise on the accuracy of detecting particulate matter using synthetic data. It varies focus blur parameters in three settings to mirror real-world conditions, adjusting the focus area from 100 to 70 and blur strength from 1 to 4. Figure 7, Figure 8, Figure 9, Figure 10 and Figure 11 delineate the comparative performance of particle detection under these input parameters. These changes simulated the effects of environmental factors such as lighting and camera stability on image quality. Results showed that increasing blur strength and decreasing focus area significantly reduced the detection accuracy of $PM_{10}$ particles, emphasizing the importance of optimal photograph conditions for effective particulate matter monitoring. This underscores the need to capture good-quality images to improve the reliability of sensor-based environmental assessments. Figure 8 shows the accuracy of the proposed method with the addition of focus blur noise for varying input parameters.

### 4.5. Examining the Effects of Adding White Balance Adjustment Noise in Synthetic Data Samples

In this experimentation, the addition of white balance adjustment noise is evaluated to improve the realism of synthetic $PM_{10}$ particle detection images, intended to resemble those taken by mobile phones. Figure 9 presents a comparative analysis of detection accuracy across varying levels of white balance noise with temperature adjustments from −1 to −20. The data reveal that the accuracy without noise remains consistently high across different particle counts. With the addition of white balance noise at Temp = −1, there is a noticeable decrease in accuracy as particle counts increase, with relatively high accuracy at lower concentrations, diminishing somewhat at higher concentrations (from 1.00 for 100 particles to 0.926 for 10,000 particles). A more substantial decrease in accuracy is apparent with greater white balance noise levels (Temp = −20), where the reduction is evident even at lower particle counts. This analysis demonstrates how different levels of simulated white balance noise can impact the accuracy of $PM_{10}$ particle detection, offering essential insights into the optimization of synthetic image generation for realistic simulation and effective Citizen Science initiatives in PM particle monitoring.

### 4.6. Examining the Effects of Adding Gaussian Noise and White-Balanced Noise in Synthetic Data Samples

The impact of introducing Gaussian noise and white balance adjustments were explored together on synthetic data samples for $PM_{10}$ particle detection. The Gaussian noise was parameterized by mean and variance levels, while the white balance adjustment was added through temperature changes. These noises were added to the image samples to simulate realistic environmental conditions that could affect the accuracy of particle detection technologies. It is evident that the accuracy levels remain relatively high, underscoring the robustness of the image processing technique employed. The analysis conclusively demonstrates that our image processing approach maintains a commendable performance even when subjected to significant levels of both Gaussian noise and white balance adjustments. This experiment not only highlights the effectiveness of the current method in handling noise but also provides insights into the potential need for further optimization to enhance performance in even more challenging noisy environments. Figure 10 shows us the accuracy of the proposed method with the addition of Gaussian noise and white balance noise together for varying input parameters.

### 4.7. Examining the Effects of Adding Gaussian Noise, Focus Blur, and White-Balanced Noise in Synthetic Data Samples

The aim was to assess the robustness of the image processing techniques under challenging noise conditions. Three types of noise—Gaussian, focus blur, and white balance adjustments—were simultaneously integrated into synthetic data samples to represent $PM_{10}$ particle detection. This experiment was designed to simulate real-world scenarios where such noises might affect the accuracy of particle detection systems. Gaussian noise was parameterized by its mean and variance, focus blur was manipulated through strength and area settings, and white balance was set through temperature changes. These elements were systematically varied to observe their individual and combined effects on detection accuracy.

Even as noise parameters intensify—illustrated by increasing Gaussian noise levels, more white balance settings, and increased focus blur effects—the proposed method shows an ability to still accurately detect $PM_{10}$ particles, albeit that it slightly reduced accuracy. Figure 11 shows us the comparison of the accuracy of the proposed method with Gaussian noise, white balance noise, and focus blur noise together with varying input parameters.

## 5. Discussion

The integration of Gaussian noise into synthetic samples, with mean values ranging from 1 to 5 and variances from 4 to 8, reveals a consistent detection accuracy across varying $PM_{10}$ particle concentrations. Despite the fact that higher noise levels lead to a slight decrease in accuracy, the robustness of the detection algorithm remains commendable. This suggests that the image processing technique can effectively handle realistic noise conditions commonly encountered in mobile imaging.

Adding white balance noise, with input parameters ranging from −1 to −20, results in a subsequent decrease in accuracy as the parameter deviation increases, especially at higher particle concentrations. This underscores the sensitivity of $PM_{10}$ detection accuracy to white balance adjustments and highlights the need for careful calibration of smart phones while taking images from smartphones to participate in this project.

The application of focus blur noise, particularly with increased blur strength and reduced focus area, significantly impacts the accuracy of $PM_{10}$ detection. Higher blur strengths and smaller focus areas substantially reduce detection capabilities, stressing the importance of maintaining optimal focus in environmental sensor applications.

By combining Gaussian noise and white balance adjustments in synthetic samples, it was found that their combined effect does not lead to a significantly higher degradation of accuracy. This outcome is crucial as it simulates the types of noise commonly found in real-world images captured by smartphones. The image processing technique efficiently manages these noises by varying input parameters, thus maintaining robust performance. This capability to uphold accuracy despite multiple noises supports the practical application of the detection of $PM_{10}$ particles in real-world conditions, closely mimicking the challenges encountered in environmental monitoring.

The incorporation of all three noises—Gaussian, white balance, and focus blur—into the synthetic samples shows that focus blur most significantly impacts performance, particularly when combined with other noise types. Despite this, the methodology still achieves reasonable accuracy, suggesting potential for the detection of $PM_{10}$ particles using image processing techniques in mobile devices, where varying environmental conditions and camera qualities could introduce similar noise characteristics. This aligns with the need for robust environmental monitoring tools that can perform reliably in diverse and challenging conditions typically encountered in Citizen Science projects.

### Limitations

In this study, a low-cost image sensor was employed to detect and count particulate matter, demonstrating potential for raising awareness about air pollution among young citizens. The air meter showed a moderate correlation with $PM_{10}$ concentrations, with a coefficient of dispersion of 0.4 [21], and this indicates inherent limitations in the method’s accuracy and reliability. The interpolation method used in generating synthetic images can blur details, complicating the detection and classification of particulate matter by the proposed algorithm. Additionally, while realistic noise variations were introduced to the synthetic images, they cannot fully replicate the diversity and unpredictability of real-world conditions, potentially affecting the model’s transferability and generalization. Furthermore, the placement of image sensors outdoors exposes them to environmental factors like rain. This exposure can cause the loss of particulate matter, resulting in data inaccuracies and disparities when compared to more reliable, laser-based sensors such as the AtmoTube, which operates using laser technology and does not face issues related to moisture exposure and particle loss [43]. These environmental vulnerabilities underscore the challenges of using low-cost sensors for reliable air quality monitoring.

## 6. Conclusions and Future Work

This work has introduced an advanced image processing methodology to enhance the detection of particulate matter over petroleum-jelly-covered “paper sensors”, achieving a commendable 90% accuracy in various testing setups. The image processing process involves critical preprocessing steps such as converting images to grayscale, applying Gaussian Blur, and adaptive thresholding. These steps are pivotal for transforming pixel measurements into micrometres and ensuring accurate particle counting. To address the challenge of data scarcity—a significant barrier in precise $PM_{10}$ particle detection—the focus of this research work has been to produce a novel approach to generate synthetic images. These images, modified by parameters like resolution and particle count, incorporated complexities like Gaussian noise and focus blur to closely replicate actual environmental conditions. These realistic variations in the synthesized images have been proven not to affect the accuracy of the devised image processing sensor for particle matter. Hence, it can be stated that this work provides a low-cost, innovative air quality meter which uses petroleum jelly to capture particulate matter. This setup should reduce the current reliance on costly sensors and makes air quality monitoring more accessible, thereby democratizing environmental surveillance. This integration not only facilitates an accurate assessment of pollution levels but also enhances the scalability of the proposed method. In conclusion, this study delineates a robust, affordable sensor system using image processing techniques to detect particulate matter, promoting a deeper public understanding of air pollution challenges. By incorporating educational elements and encouraging public engagement, the methodology fosters a comprehensive approach to environmental monitoring, aiming to enhance technological proficiency and active participation in pollution mitigation among communities.

## Figures and Tables

**Table 1:** Comparison of particulate matter detection methods.

Detection Technique	Benefits	Drawbacks	Sensor Features
Tapered Element Oscillating Microbalance (TEOM)	Highly precise real-time data; automated data processing.	Requires heating, sensitive to environmental changes.	Direct mass measurement using oscillating tapered tube.
Beta Attenuation Monitors (BAMs)	Precise real-time PM monitoring; widely recognized for regulatory compliance.	Uses radioactive sources; sensitive to air moisture.	Continuous tape system; measures particle mass via beta ray attenuation.
Black Smoke Method	Simple, cost-effective; easy setup and maintenance.	Less precise; manual operation and frequent maintenance needed.	Traditional method using filter collection and weighing of black smoke.
Optical Analyzers	Real-time data; portable; high sensitivity.	Affected by external conditions; indirect mass estimation.	Utilizes light scattering and image processing for real-time analysis.

**Table 2:** Overview of sensors for particulate matter measurement [36].

Particle Matter	Sensing Module	Measurement Technique	Cost
$PM_{2.5}$	BAM-1020 Beta Attenuation Measurement Module	Beta Attenuation	Very High
	Aerocet 831 aerosol mass measurement module	Light Scattering	High
	OPC-N2 particle sensor	Light Scattering	Average
	Dn7c3ca006 $PM_{2.5}$ Unit	Light Obscuration	Low
$PM_{10}$	602 Betaplus	Beta Attenuation	Very High
	Aerocet 831 aerosol mass monitor	Light Scattering	High
	OPC-N2 particle sensor	Light Scattering	Average
	Gp2y1010au	Light Obscuration	Low

**Table 3:** Parameters for synthetic data generation.

Parameter	Description
Particle Size	Defines the fineness of dust particles, ranging from 1 to 10 mm.
Image Height and Width	Determines the dimensions of the synthetic images.
Image Resolution	Measured in pixels per micrometre, set at 1 pixel per micrometre.

**Table 4:** Correlation between dot density and air pollution level.

Description	Dots per cm^2^	Air Pollution Level
The paper has many black and grey dots. Large parts of the paper have turned grey.	>50	Very high
The paper has quite a few black and grey dots. There are some parts on the paper that have turned grey.	26–50	High
The paper has black and grey dots all over the surface, but there are no fields that are completely grey.	11–25	Medium
The paper has only a few black and grey dots, and there are no fields that are completely grey.	<11	Low

## Data Availability

The data presented in this study are openly available in Zenodo at https://doi.org/10.5281/zenodo.13887798.

## Appendix A. Particle Size Conversion: Pixels to Micrometres

The realm of microscopy, particle imaging and the ability to correlate the dimensions observed in a digital image with real-world metrics are of paramount importance. Digital images inherently represent any captured object in terms of pixels, which are the smallest addressable elements in an image. Each pixel represents a certain area or length in the actual sample, depending on the resolution of the capturing device. The challenge lies in bridging the gap between these pixel-based measurements and real-world micrometre-based measurements, especially when examining micro-sized particles or structures.

This was achieved by first determining the real-world dimensions of the image sample. The size of the inner square is 6 cm by 6 cm. In the air meter from NILU, the measuring part consists of a square of 6 cm for each side, so for finding the area in centimeters of the region of interest the side was squared. Next, digital dimensions of the image were evaluated. This refers to the total number of pixels that make up the width and height of the image. By multiplying these two values, the total number of pixels in the area of the image was calculated.

(A1) total_pixels=width×height

With both the real-world area in μm^2^ and the digital image area in pixels, a conversion factor or resolution can be deduced. This resolution, defined as pixels per micrometre (ppm), is derived by dividing the total pixel area by the real-world area in μm^2^. To convert this area-based resolution to a linear one, a square root operation is employed.

(A2) $$\mathrm{resolution}\_\mathrm{area}\_\mathrm{based}=\frac{\mathrm{total}\_\mathrm{pixels}}{\mathrm{real}\_\mathrm{world}\_\mathrm{area}\_\mu m}$$

With this conversion factor, any particle’s size in pixels can be translated to its actual size in micrometres.

Equations (A1) and (A2) explain the basic steps involved in converting from pixels to micrometres. As a first step, the focus is directed towards each closed contour within the image. These contours symbolize particles or distinct entities. For every contour, the encompassed area is calculated, which represents the sum of pixels it contains. The formula for the area of a circle, $A=\pi r^{2}$, is employed. Here, *A* signifies the area and *r* designates the radius. Manipulating this formula, the radius and subsequently, the diameter *d* (being 2r) of the particle can be derived using

(A3) $$d=\sqrt{\frac{4A}{\pi }}$$

Thus, each contour’s area is transposed into a diameter represented in pixels.

To properly contextualize this diameter in terms of real-world dimensions, particularly in micrometres, it is necessary to undertake a conversion. Leveraging the previously deduced ’pixels per micrometre’ (ppm) as the conversion metric, the diameter in pixels is divided by ppm to yield the diameter in micrometres:

(A4) $$d_{\mu m}=\frac{d_{\mathrm{pixels}}}{\mathrm{ppm}}$$

Equations (A3) and (A4) demonstrate the formula to find the diameter of contours. The culmination of this procedure is a comprehensive list of diameters, each corresponding to a detected particle and articulated in micrometres. This list stands as a tangible metric, facilitating a deeper examination of particle sizes in relation to their real-world dimensions.

### Classification of Particulate Matter

For the $PM_{10}$ particle detection and classification, a synthetic dataset was employed to analyze the efficacy of an image processing-based algorithm functioning as a cost-effective methodology for air pollution monitoring. The first step was to detect the $PM_{10}$ particles inside the synthetic images. Following this initial step, noise was meticulously removed from the images through preprocessing techniques. Subsequently, features were extracted by calculating the contours and their respective diameters in pixels, which were then converted from pixels to micrometres to ensure accuracy in measurement. Based on these diameters, a conditional classification system was applied, where particles with diameters of 10 mm or less were classified as $PM_{10}$. This systematic approach underscores the potential of image processing technologies in environmental monitoring using low-cost sensors.

(A5) $$\mathrm{Classification}=\left\{\begin{array}{cc} {\mathrm{PM}}_{10} & \mathrm{if}\;\mathrm{Diameter}\leq 10\;\mathrm{mm}\\ \mathrm{Not}{\mathrm{PM}}_{10} & \mathrm{if}\;\mathrm{Diameter}>10\;\mathrm{mm} \end{array}\right\}$$

## Appendix B. Additional Details on Image Resizing Algorithm

This section provides the detailed pseudocode and matrix operations used in the image resizing process described in Section 3. The pseudocode illustrates the steps taken to resize an image based on matrix operations and the Gauss–Jordan elimination method.

### Matrix Operations and Pseudocode for Upscaling of Image

Input: Original Image Output: Resized Image

(A6) $$W_{-1}\left(S\right)=-S^{3}+2S^{2}-S$$

(A7) $$W_{0}\left(S\right)=3S^{3}-5S^{2}+2$$

(A8) $$W_{1}\left(S\right)=-3S^{3}+4S^{2}+S$$

(A9) $$W_{2}\left(S\right)=S^{3}-S^{2}$$

**Algorithm A1** Algorithm for resizing an image
1: **for** x=1 **to** image width in pixels **do** 2: **for** y=1 **to** image height in pixels **do** 3: Arrange augmented y matrix using the weights from the referenced equations: $$\left[\begin{array}{cccc} 1 & y_{1} & y_{1}^{2} & y_{1}^{3}\\ 1 & y_{2} & y_{2}^{2} & y_{2}^{3}\\ 1 & y_{3} & y_{3}^{2} & y_{3}^{3}\\ 1 & y_{4} & y_{4}^{2} & y_{4}^{3} \end{array}\right]\left[\begin{array}{c} W_{-1}\left(S_{y}\right)\\ W_{0}\left(S_{y}\right)\\ W_{1}\left(S_{y}\right)\\ W_{2}\left(S_{y}\right) \end{array}\right]$$ ▹*W* are the weight functions and $S_{y}$ is the scaling factor in y direction. 4: Apply the Gauss Jordan elimination method to solve for *p* values by Equation (A7): $$p=W_{-1}\left(S_{y}\right)f_{i-1,j}+W_{0}\left(S_{y}\right)f_{i,j}+W_{1}\left(S_{y}\right)f_{i+1,j}+W_{2}\left(S_{y}\right)f_{i+2,j}$$ 5: Enter the y value of the pixels 6: Arrange augmented x matrix using the weights from the referenced equations: $$\left[\begin{array}{cccc} 1 & x_{1} & x_{1}^{2} & x_{1}^{3}\\ 1 & x_{2} & x_{2}^{2} & x_{2}^{3}\\ 1 & x_{3} & x_{3}^{2} & x_{3}^{3}\\ 1 & x_{4} & x_{4}^{2} & x_{4}^{3} \end{array}\right]\left[\begin{array}{c} W_{-1}\left(S_{x}\right)\\ W_{0}\left(S_{x}\right)\\ W_{1}\left(S_{x}\right)\\ W_{2}\left(S_{x}\right) \end{array}\right]$$ ▹*W* are the weight functions and $S_{x}$ is the scaling factor in x direction. 7: Apply the Gauss Jordan elimination method to solve for *p* values by Equation (A9): $$p=W_{-1}\left(S_{x}\right)f_{i,j-1}+W_{0}\left(S_{x}\right)f_{i,j}+W_{1}\left(S_{x}\right)f_{i,j+1}+W_{2}\left(S_{x}\right)f_{i,j+2}$$ 8: Enter the x value of the pixels to searched equation 9: **end for** 10: **end for** 11: Perform rounding on each pixel value
